# Continued importance in translation from research to health policy in China

**DOI:** 10.1186/s12967-016-1021-7

**Published:** 2016-09-20

**Authors:** Jun Zhang, Fan Jiang, Xiaoming Shen

**Affiliations:** 1 Shanghai Jiao Tong University School of Public Health, Shanghai, China; 2 Ministry of Education-Shanghai Key Laboratory of Children’s Environmental Health, Xinhua Hospital, Shanghai Jiao Tong University School of Medicine, Shanghai, 200092 China; 3 Ministry of Education-Shanghai Key Laboratory of Children’s Environmental Health, Shanghai Children’s Medical Center, Shanghai Jiao Tong University School of Medicine, Shanghai, China; 4 Ministry of Education-Shanghai Key Laboratory of Children’s Environmental Health, Xinhua Hospital and Shanghai Children’s Medical Center, Shanghai Jiao Tong University School of Medicine, Shanghai, 200092 China

**Keywords:** Precision medicine, Translation, Research, Health policy

## Abstract

Precision Medicine may be considered as another strategic effort of “from-bench-to-beside” translation. In order to have a maximal impact on population health, the translation must go further. A new translational medicine paradigm is proposed to improve the clarity of conceptual pathways and facilitate translation from research to health policy. The latter is particularly important in low- and middle-income countries where the need to improve population health is immediate and immense.

The Precision Medicine Initiative proposed by the president of the United States (U.S.) last year has swept through the research landscape in China. It is considered by some as a new direction of future research and a new model for health care delivery [1]. The National Health and Family Planning Commission of China reportedly plans to spend 60 billion Ren Min Bi (RMB) (US$9.2 billion) in Precision Medicine in the next 15 years [2].

At the core of the Precision Medicine Initiative lies the proposal to use (1) large databases such as the human genome sequence; (2) individual characterizing information from modern technologies such as proteomics, metabolomics, genomics, and diverse cellular assays; (3) computational tools for analyzing big data; and (4) mobile health technology, to prevent and treat diseases by taking variability of individual patients into account [3]. The concept is noble but to implement this strategy requires seas of knowledge. Precision testing alone is insufficient; making clinical sense of the testing results, counseling patients, judging clinical actionability and providing clinical guidance to patient care are essential. To this end, the Initiative plans to launch a large prospective cohort study of over 1 million Americans to obtain necessary information and translate it into clinical practice. Recent requests for proposals from the Ministry of Science and Technology of China followed suit. In addition to a million-people cohort, China goes further to establish more than a dozen of disease-specific cohorts with a focus on treatment and prognosis research. If translational medicine emphasizes the process from research to practice to policy, Precision Medicine is a method of research and practice. In essence, Precision Medicine is another strategic effort of from-research-to-practice translation.

But the translation must go further. If the ultimate goal of translational medicine is to improve health of the population, translation from research to prevention and health policy remains to be of critical importance, especially in low- and middle-income countries [4]. We hereby propose a new paradigm that hopefully can improve the clarity of conceptual pathways and facilitate translation from medical research to health policy (Fig. 1).

**Fig. 1:**
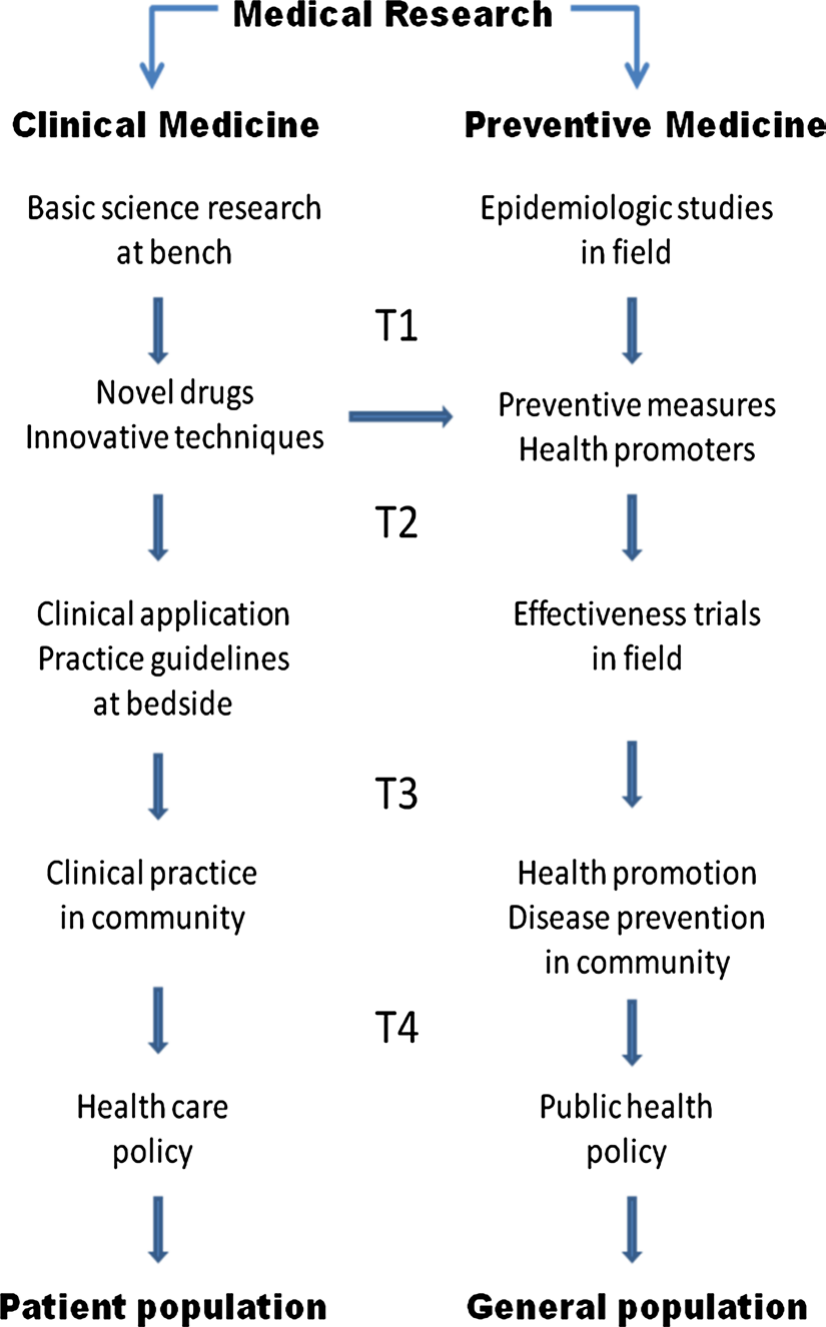
A new translational medicine paradigm. Steps in translational medicine

There are two similar but distinct pathways in translational research. The clinical pathway has been well characterized in the past decade and the importance of translating basic science discoveries into clinical application and from clinical practice to health care policy, eventually benefitting a large population has been demonstrated in a number of successful examples in China [5]. However, the preventive medicine pathway is much less well conceptualized and popularized. Epidemiologic studies identify risk factors for diseases (e.g., smoking) or health promoters (e.g., exercise). Health promoters can also come from basic and/or clinical research, e.g., vaccines. Through rigorous testing, most often by randomized field trials, effective preventive measures and health promoters are established. Yet, implementation of preventive strategies in community also requires deep knowledge in health care delivery and disease prevention systems, health economics, health education and behavior modification, and even relevant regulation and law. T3 is a process of knowledge acquisition through social and behavioral studies and knowledge transfer from academic setting to community. If a preventive measure can be successfully implemented and demonstrate its effectiveness, setting up a corresponding public health policy should be advocated (T4).

In the context of Precision Medicine, certain cancer treatments have taken the lead in the clinical pathway and the treatment success was improved substantially [6]. Yet, much research is needed particularly in treating common diseases, which will have much greater implications in health care policy. More importantly, and probably more difficult as well is to establish personalized prevention. Evidence indicates that for common and complex diseases such as hypertension, genetics accounts for a small proportion of the etiology; environment and behavioral factors are the main determinants of such diseases. Thus, while genetic information is helpful, available literature does not seem to lend a strong support yet for a policy of universal gene sequencing for the purpose of prevention, despite that the cost of whole genome sequencing has dropped substantially. The interpretation of genome sequencing results is another significant challenge. The prevention pathway of the translational medicine framework remains as an important process to translate from research to public health policy in China in the near future.

In spite of some successes in our own practice in policy translation [7], an efficient and effective channel and applicable process to transfer knowledge from academicians to policy makers have not been established in China; nor is it being systematically practiced. Most physicians and scientists feel that T4 is out of their reach and, therefore, may not have the desire to carry out advocacy. Policy makers, on the other hand, often have multiple issues at hand that compete for their attention. Evidence shows that it is critical to build and maintain a good relationship and trust between researchers and policy makers [8]. Policy makers also rank direct interaction with researchers as the most effective way to take up science into policy [9]. It is beneficial for researchers to inform and engage policy makers in research process as early as possible and throughout the study period. In addition, policy makers need synthesized evidence that is easy to understand and communicate [8]. Consequently, “knowledge broker” or “professional communicator” model was proposed to facilitate the communications between researchers and policy makers [10].

China offers great opportunities to apply evidence-based policy to the field because it is undertaking tremendous infrastructure and capacity building as well as system reforms. An individual scientific discovery may be translated or localized into one particular policy. Multiple scientific advances may be embedded in one comprehensive system reform. And a large national or city project may incorporate a number of health policies and scientific innovations.

To achieve these requires both researchers and policy-makers to change their mindset, value and invest in translation research. Funding agencies in North America and Europe often explicitly require plans and tangible measures for knowledge translation and policy implications in grant applications in recent years. A number of centers for translation research were funded by the National Institutes of Health (NIH) across the U.S. Within NIH a Center for Translation Research and Implementation Science was established last year [11]. These models and, more importantly, their contents deserve a careful assessment in China. We would further suggest that local governments may also consider supporting translation research centers with T4 translation as part of the mandate to address urgent local needs. Policy makers can solicit ideas from researchers to solve local problems. A review board may select promising candidates and communicate with policy makers for further consideration. When a sound health policy is successfully translated and effectively applied, the results can be especially rewarding in China in terms of the scale of impact and the magnitude of change in a relatively short time [7].

In summary, the Precision Medicine Initiative propels the translational medicine movement to another height. For a country like China, which accounts for 20 % of the world’s population, the need to improve population health is immediate and immense. It will be imperative to maximize the benefits of the discoveries by Precision Medicine, not only for treatment but, more importantly, for disease prevention. When we try to advance Precision Medicine, the bigger picture of Translational Medicine, particularly the translation from research to health policies, should not be forgotten.

## Abbreviations

U.S.: The United States
RMB: Ren Min Bi
NIH: National Institutes of Health
